# Lead-induced Hepatotoxicity and Evaluation of Certain Anti-stress Adaptogens in Poultry

**DOI:** 10.4103/0971-6580.75866

**Published:** 2011

**Authors:** M. Ratan Kumar, K. S. Reddy, A. Gopala Reddy, Rajasekhar A. Reddy, Y. Anjaneyulu, Dilip G. Reddy

**Affiliations:** Department of Pharmacology and Toxicology, College of Veterinary Science, Rajendranagar, Hyderabad - 500 030, Andhra Pradesh, India; 1 Department of Pathology, College of Veterinary Science, Korutla, Andhra Pradesh, India; 2 Department of Pathology, College of Veterinary Science, Rajendranagar, Hyderabad - 500 030, Andhra Pradesh, India

**Keywords:** ATPase, broilers, CYP_450_, lead, hepatotoxicity

## Abstract

A total of 225 day-old sexed male broiler chicks (*Vencobb* strain) were divided randomly into 15 groups consisting of 15 chicks in each group to study the toxicity of lead on hepatocytes. Group 1 was maintained on basal diet, group 2 on polyherbal formulation (PHF; stressroak), group 3 on shilajith, group 4 on amla and group 5 on vit E + Se. Group 6 was maintained on lead for 6 weeks and group 7 on lead for 4 weeks and subsequently on basal diet without lead for the remaining 2 weeks. Groups 8, 9, 10 and 11 were given lead along with PHF, shilajith, amla and vit E + Se, respectively, throughout 6 weeks. Groups 12, 13, 14 and 15 were given lead containing diet for the first 4 weeks and subsequently treated with PHF, shilajith, amla and vit E + Se, respectively, for the remaining 2 weeks. The activity of alanine transaminase (ALT) was significantly (*P*<0.05) increased in the toxic control groups at the end of 4^th^ week as compared to group 1. However, following treatment, there was a significant (*P*<0.05) reversal in groups 12–15. The activity of Na^+^/K^+^-ATPase, Ca^2+^ATPase, Mg^2+^ATPase and CYP_450_ was significantly (*P*<0.05) reduced in the liver of toxic control groups 6 and 7 as compared to groups1 through 5, which had the maximum activity of all the groups. Groups 8 through 15 revealed a significant (*P*<0.05) increase in the activity of these hepatocytic enzymes. The histological sections of the liver in lead toxic control (group 6) showed moderate focal lymphoid aggregates in liver, whereas the lesions were mild to moderate in treated groups and there were no observable lesions in plain control groups. The study revealed protective effect of PHF (stressroak), shilajith, amla and vit E + Se in lead-induced hepatocytic damage.

## INTRODUCTION

Lead is one of the widely dispersed toxic substances and lead gasoline combustion in vehicles has accounted for as much as 90% of the total anthropogenic sources of environmental lead.[1] Lead produces acute and chronic poisoning and induces a broad range of physiological, biochemical and behavioral dysfunctions resulting in reduced performance and death in livestock. Lead affects the metabolism of other minerals and has affinity for bone, where it acts by replacing calcium. Thus, the highest concentrations of lead are usually found in bone, kidney and liver.[2] Lead is toxic to poultry at much lower levels than previously recognized.[3] Renal activities of Na^+^/K^+^-ATPase and K^+^-dependent phosphatase were decreased to 50–70% of control values by long-term (12 weeks) ingestion of lead in rats.[4] *In vitro* lead at 50 and 100 *μ*M significantly inhibited ATPase activity in all regions of the rat brain.[5] However, literature is scanty on the effect of lead on the hepatocytic membrane ATPases and the influence of adaptogens on lead-induced derangement of ATPases. Keeping the above facts in view, the present study was taken up to examine the effect of lead on hepatic enzyme profile and histology, and to assess the ameliorating effect of certain adaptogens and polyherbal formulations (PHFs).

## MATERIALS AND METHODS

A total of 225 sexed male broiler chicks (*Cobb* strain) of day-old age were randomly divided into 15 groups consisting of 15 chicks in each. Feed and water were provided *ad libitum* throughout the experiment. Groups 1, 2, 3, 4 and 5 were maintained on basal diet, PHF (stressroak; Ayurvet Ltd., Himachal Pradesh; composition: *Withania somnifera, Ocimum sanctum, Phyllanthus emblica, Mangifera indica* and shilajith at 100 ppm in feed), shilajith (100 ppm in feed), amla (500 ppm in feed) and vit E + Se (300 ppm + 0.3 ppm in feed), respectively, and groups 6 and 7 were the toxic controls that were kept on lead for 6 and 4 weeks, respectively. Groups 8, 9, 10 and 11 were given lead along with PHF, shilajith, amla and vit E + Se, respectively, for 6 weeks. Groups 12, 13, 14 and 15 were maintained on lead for the first 4 weeks and on PHF, shilajith, amla and vit E + Se, respectively, for the subsequent 2 weeks.

Blood samples were drawn from the wing vein at the end of 4^th^ and 6^th^ weeks for the assay of alanine transaminase (ALT), using commercial diagnostic kits (Qualigens Pvt. Ltd., Mumbai, India). For preparation of liver microsomes,[6] liver pieces were collected in cold conditions by using pre-cooled petri dishes and were blotted free of blood and tissue fluid. After being weighed, the pieces were cross-chopped with surgical scalpel into fine slices, chilled in ice-cold 0.25 M sucrose and then blotted on a filter paper. The tissues were minced and homogenized in 10 mM Tris-HCl buffer (pH 7.4) at a concentration of 10% w/v with 25 strokes of tight Teflon homogenizer at a speed of 2500 rpm. The prolonged homogenization under hypotonic condition disrupted the ventricular structure of cells so as to release soluble proteins and leave membrane and non-vascular matter in sediment form. The homogenates were centrifuged at 10,000× *g* at 4°C for 20 min.[7] The supernatant was re-centrifuged for 1 hour at 100,000× *g* at 4°C using high-speed cooling centrifuge. The sediment was re-suspended in Tris-HCl buffer (pH 7.4) to get the final concentration of 10% and was used for the assay of membrane-bound enzymes.

Total ATPase and Mg^2+^ATPase activities were determined by the method of Sadasivudu *et al*,[8] Na^+^/K^+^-ATPase activity was estimated as the difference between the total ATPase and Mg^2+^ATPase. Ca^2+^ATPase activity was determined by the method described by Kodama *et al*.[9]

For CYP assay, livers were removed rapidly, weighed and perfused with ice-cold 1.15% KCl solution containing 0.05 M ethylenediaminetetraacetic acid (EDTA). Each of the perfused livers was homogenized with two volumes of ice-cold 0.25 M sucrose in the homogenizer. The homogenate was centrifuged at 10,000× *g* for 30 min in a refrigerated centrifuge. Microsomes from the supernatant fraction were isolated by the procedure of Cinti *et al*,[10] The microsomal pellet was washed once with 1.15% KCl solution containing 0.05 mM EDTA, re-suspended in phosphate buffer (pH 7.4) and the suspension was used for microsomal enzyme assays.[11] Total protein was estimated by the method described by Lowry *et al*,[12] Microsomal suspension in phosphate buffer (1.0 mg protein/ml) was treated with a few grains of sodium dithionate and taken in the reference and sample cuvettes and the baseline was recorded from 400 to 510 nm. The contents of the sample cuvette were transferred into a tube and bubbled gently with CO for 60 seconds. Then, the contents were transferred back to the sample cuvette and the difference in spectrum was recorded from 400 to 510 nm again. The difference in absorbance between 450 and 490 nm was then used for the calculation of CYP_450_content using the extinction coefficient difference (ΔE_450-490_ nm of 91 cm/mM).[13]

The data were subjected to statistical analysis by applying two-way analysis of variance (ANOVA). Differences between means were tested using Duncan’s multiple comparison test and significance was set at *P*<0.05.

## RESULTS AND DISCUSSION

**Figure 1:**
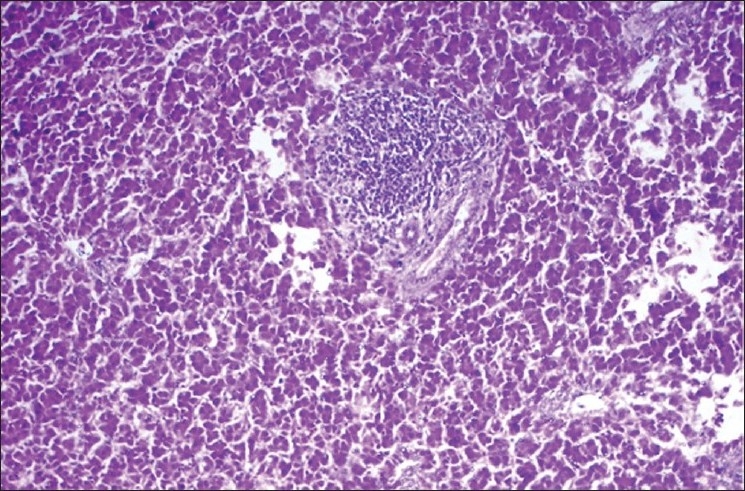
Liver of group 6 showing moderate focal lymphoid aggregates (H and E ×100)

**Figure 2:**
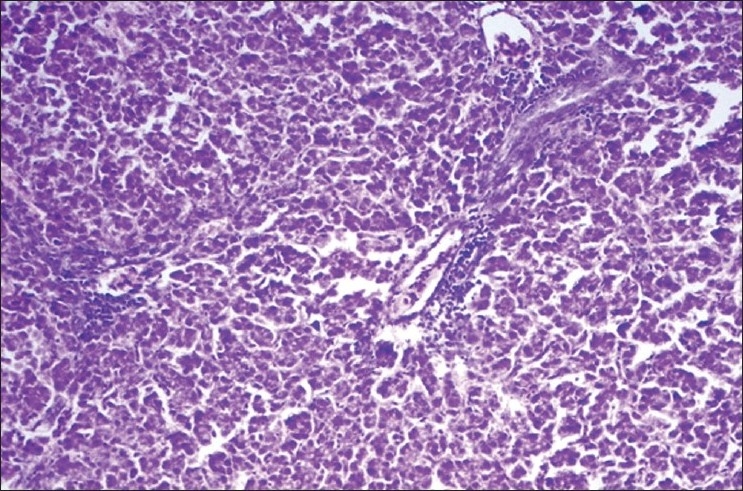
Liver of group 11 showing mild central vein congestion and paracentral infiltration

The activity of ALT (units/l) in basal diet control (group 1) was 19.217±2.156, which was significantly (*P*<0.05) elevated in lead toxic control groups 6, 7, 12, 13, 14 and 15 at the end of 4^th^ week. The activities of therapeutic control groups 2–5 were comparable to that of group 1 on 4^th^ and 6^th^ weeks. The groups 8–11 revealed a significant (*P*<0.05) increase in the activity of ALT at the end of 4^th^ and 6^th^ weeks as compared to group 1 and other therapeutic controls (groups 2–5) at respective time intervals. In groups 12–15, there was a significant (*P*<0.05) decrease in the activity at the end of 6^th^ week as compared to groups 6 and 7 [Table 1]. The histological sections of the liver from the lead toxic control (group 6) showed moderate focal lymphoid aggregates [Figure 1], while group 11 showed mild central vein congestion and paracentral infiltration [Figure 2]. The lesions were very mild or absent in treated groups 12 through 15 and plain controls.

**Table 1:** Activity of ALT and CYP_450_ in different groups of broiler chicks

Group	ALT (IU/l)		CYP_450_ (nmol/mg microsomal protein)
	4^th^ week	6^th^ week	6^th^ week
Basal diet (1–42 days)	19.217±2.156^cA^	24.962±1.453^bA^	0.2520±0.009^d^
PHF (stressroak) (1–42 days)	13.588±1.737^aA^	20.712±1.015^aA^	0.2489±0.004^d^
Shilajith (1–42 days)	15.306±0.755^abA^	21.185±0.940^aA^	0.2529±0.010^d^
Amla (1–42 days)	17.433±0.928^bcA^	21.807±0.808^aA^	0.2510±0.008^d^
Vit E + Se (1–42 days)	16.906±1.15^bcA^	23.236±0.981^abA^	0.2499±0.010^d^
Lead (1–42 days)	60.114±3.110^hA^	69.082±1.671^jA^	0.1262±0.009^a^
Lead (1–28 days); basal diet (29–42 days)	60.531±1.481^hA^	62.023±0.536^fgA^	0.1523±0.011^ab^
Lead + PHF (stressroak) (1–42 days)	50.160±1.076^dA^	61.345±1.113^fA^	0.1850±0.007^bc^
Lead + shilajith (1–42 days)	52.941±1.236^eA^	64.278±1.374^ghA^	0.1890±0.010^c^
Lead + amla (1–42 days)	55.340±0.796^efA^	66.061±1.026^hiA^	0.1869±0.010^c^
Lead +Vit E + Se (1–42 days)	57.079±0.871^fgA^	67.164±1.922^ijA^	0.1820±0.006^bc^
Lead (1–28 days); PHF (stressroak) (29–42 days)	59.028±1.539^ghA^	32.453±1.609^cA^	0.1922±0.0155^c^
Lead (1–28 days); shilajith (29–42 days)	58.250±1.341^ghA^	35.226±0.981^dA^	0.1912±0.009^c^
Lead (1–28 days); amla (29–42 days)	58.929±1.988^ghA^	37.344±1.182^deA^	0.1890±0.003^c^
Lead (1–28 days); vit E + Se (29–42 days)	59.115±0.663^ghA^	37.767±1.062^eA^	0.1932±0.007^c^

Values are mean±SE of eight observations. Means with different alphabets as superscripts differ significantly (*P*<0.05); ANOVA. Capital alphabets (horizontal comparison); small alphabets (vertical comparison); ALT, alanine transaminase

The activity of ALT was determined to assess the degree of damage to liver as the levels of certain enzymes like ALT, aspartate transaminase (AST), gamma glutamyl transferase (GGT), etc., are elevated following hepatocellular injury.[14] In this study, the activity of ALT was significantly elevated in the lead toxic control group, suggesting the hepatocellular insult following administration of lead. These results are further substantiated from the histopathological studies of liver, which revealed moderate focal lymphoid aggregates in lead toxic control. Treatment with PHF (stressroak), shilajith, amla and vit E + Se, following discontinuation of lead resulted in significant reduction in the activity of ALT. The hepatocellular injury due to lead could be attributed to the lead-induced generation of reactive oxygen species (ROS) or free radicals and the reversal of the findings following treatment could be attributed to the antioxidant and the hepatoprotective potential of the drugs in test. Alcoholic and aqueous extracts of the fruits of *Emblica* have shown hepatoprotective properties in experiments in rats.[15] Further, the treatment controls, which were not exposed to lead did not manifest any significant change in the activity of ALT, and also there were no significant lesions in the liver tissues. However, simultaneous supplementation of drugs in the test along with lead revealed a significant increase in ALT activity at different time intervals when compared to the plain control groups, though the values were significantly lower in comparison to pure lead toxic control groups 6 and 7. This finding suggests the prophylactic potential of these drugs to prevent lead-induced toxic manifestations, though there was no complete prevention of changes.

The CYP_450_ activity (nmol/mg microsomal protein) in basal diet control (group 1) was 0.2520±0.009, which was significantly (*P*<0.05) decreased in lead toxic control groups 6 and 7 at the end of 6^th^ week. The activities of therapeutic control groups 2–5 revealed no significant difference in CYP_450_ activity as compared to group 1, while the groups 8–11 revealed a significant (*P*<0.05) decrease in the activity as compared to group 1, and groups 12-15 revealed a significant (*P*<0.05) increase in the activity as compared to group 6 at the end of 6^th^ week [Table 1].

The activity of Na^+^/K^+^-ATPase, Mg^2+^ATPase and Ca^2+^ATPase (mM of Pi liberated/mg protein) in basal diet control (group 1) was 2.5919±0.062, 1.8691±0.098, and 1.6609±0.081, respectively, which was significantly (*P*<0.05) decreased in lead toxic control groups 6 and 7 at the end of 6^th^ week. The activities of therapeutic control groups 2-5 revealed no significant difference in the activity of ATPases as compared to group 1 at the end of 6^th^ week. Groups 8-11 revealed a significant (*P*<0.05) decrease in the activity as compared to group 1 at the end of 6^th^ week. Groups 12-15 revealed a significant (*P*<0.05) increase in the activity as compared to group 6 [Table 2].

**Table 2:** Activities of Na^+^/K^+^-ATPase, Mg^2+^ATPase and Ca^2+^ATPase in the liver of different groups of broiler chicks

Group	Na^+^/K^+^-ATPase (mM of Pi liberated/mg protein)	Mg^2^+ATPase (mM of Pi liberated/mg protein)	Ca^2+^ATPase (mM of Pi liberated/mg protein)
Basal diet (1–42 days)	2.5919±0.062^e^	1.8691±0.098^d^	1.6609±0.081^e^
PHF (stressroak) (1–42 days)	2.5833±0.085^e^	1.8817±0.074^d^	1.6553±0.043^e^
Shilajith (1–42 days)	2.4485±0.102^de^	1.8911±0.026^d^	1.6845±0.063^e^
Amla (1–42 days)	2.6009±0.091^e^	1.8699±0.036^d^	1.6695±0.046^e^
Vit E + Se (1–42 days)	2.6324±0.062^e^	1.8831±0.044^d^	1.6806±0.061^e^
Lead (1–42 days)	0.9713±0.045^a^	1.1284±0.054^a^	0.7723±0.037^a^
Lead (1–28 days); basal diet (29–42 days)	1.1699±0.097^ab^	1.3206±0.077^ab^	0.9886±0.042^ab^
Lead + PHF (stressroak) (1–42 days)	2.2062±0.063^cd^	1.5609±0.059^bc^	1.4574±0.091^de^
Lead + shilajith (1–42 days)	2.0524±0.121^c^	1.3261±0.052^ab^	1.2499±0.081^cd^
Lead + amla (1–42 days)	1.8905±0.076^c^	1.3296±0.107^ab^	1.2450±0.056^cd^
Lead + vit E + Se (1–42 days)	1.9141±0.069^c^	1.3519±0.078^ab^	1.2836±0.074^cd^
Lead (1–28 days); PHF (stressroak) (29–42 days)	2.1842±0.139^cd^	1.6240±0.056^cd^	1.1567±0.113^bc^
Lead (1–28 days); shilajith (29–42 days)	2.1204±0.109^cd^	1.4530±0.1196^bc^	1.0929±0.049^bc^
Lead (1–28 days); amla (29–42 days)	1.3955±0.076^b^	1.3439±0.094^ab^	1.0499±0.056^bc^
Lead (1–28 days); vit E + Se (29–42 days)	2.1639±0.159^cd^	1.5565±0.071^bc^	1.1474±0.015^bc^

Values are mean±SE of eight observations. Means with different alphabets as superscripts differ significantly (*P*<0.05); ANOVA

In this study, significant reduction in the activities of Na^+^/K^+^-ATPase, Ca^2+^ATPase, Mg^2+^ATPase and CYP_450_ in the liver of lead toxic control groups 6 and 7 may be attributed to the lipid proxidation by free radicals in lead treated groups, since these membrane-bound enzymes are SH group containing enzymes, which are lipid-dependent and their activity is likely to be altered with the change in the composition of membrane lipids.[6] The treated groups revealed slight improvement in the membrane-bound enzymes though the activities were not exactly similar to those of controls. It has been reported that dietary lead alters fatty acid composition and membrane peroxidation in chick liver microsomes.[16] These findings could be further substantiated from the results of the present study, wherein there was a significant increase in the activity of ALT (suggesting hepatocellular injury) and histopathological changes in liver in the toxic control group 6 as compared to the plain controls and treated groups.

In conclusion, the study enunciated that lead induces damage to the hepatocytes leading to elevation of ALT along with reduction in CYP and membrane ATPases. Supplementation of anti-stress agents is of use to reverse the hepatocytic damage caused by lead. Amongst the drugs in test, PHF (stressroak) was found to be superior owing to its synergistic antioxidant herbs, followed by shilajith, amla and vit E + Se in the given order.
